# Supplementary material for: A Likelihood Approach for Real-Time Calibration of Stochastic Compartmental Epidemic Models
Source: PLoS Comput Biol. 2017 Jan 17;13(1):e1005257. doi: 10.1371/journal.pcbi.1005257 (PMC5240920; doi:10.1371/journal.pcbi.1005257)
Supplement: S1 File — (TAR.GZ) [file pcbi.1005257.s014.tar.gz › HSPH_Online-SI-Revision/Additions/IPoi10/n100-extreme_noisy/n100-extreme_noisy_BENCH_table8.pdf]

1	R0	{2.76052, 16.3913}	{2.8939, 16.8013}	{{2.8939, 16.8013}, {2.8939, 16.8013}}	{4.8317, 2.50133}	inf	3200.	5814.81	{2769., 9762.}	86.4848	0.235313	newly	1200.81	2088.93	{976., 3509.}	80.2411	0.223238	Reff	0.245824	0.243842	{0.215885, 0.283573}	9.36559	ar	92 090.	93 264.4	{93 103., 93 413.}	1.27529
2	R0	{2.18202, 15.1734}	{2.86095, 19.9929}	{{2.86095, 19.9929}, {2.86095, 19.9929}}	{31.1147, 31.7628}	inf	3327.	3283.21	{1503., 6497.}	48.4287	0.202829	newly	1337.	1014.06	{466., 2021.}	50.7355	0.269111	Reff	0.385257	0.22649	{0.208821, 0.254224}	41.2107	ar	84 264.	93 028.6	{92 850., 93 201.}	10.4013
3	R0	{2.57891, 12.5971}	{2.95376, 15.278}	{{2.95376, 15.278}, {2.95376, 15.278}}	{14.5352, 21.2819}	inf	1362.	2394.07	{1095., 3309.}	84.4293	0.207115	newly	609.	930.485	{391., 1297.}	67.9344	0.178944	Reff	0.262765	0.204271	{0.191492, 0.215447}	24.1861	ar	90 302.	93 717.	{93 523., 93 866.}	3.78177
4	R0	{2.5856, 9.28508}	{2.83858, 10.6973}	{{2.83858, 10.6973}, {2.83858, 10.6973}}	{9.78419, 15.2096}	inf	433.	1469.97	{604., 2592.}	240.232	0.450615	newly	254.	752.079	{317., 1328.}	197.582	0.393332	Reff	0.254526	0.215103	{0.206876, 0.224276}	15.6046	ar	90 312.	92 822.	{92 650., 92 992.}	2.77927
5	R0	{2.4406, 12.1341}	{2.95376, 15.278}	{{2.95376, 15.278}, {2.95376, 15.278}}	{21.026, 25.9096}	inf	1891.	3606.09	{1576., 4980.}	98.3442	0.230225	newly	894.	1406.64	{619., 1898.}	75.65	0.197708	Reff	0.297534	0.217954	{0.195864, 0.224279}	31.9397	ar	88 597.	93 738.9	{93 559., 93 874.}	5.8037
6	R0	{2.72155, 15.4094}	{2.8939, 16.8013}	{{2.8939, 16.8013}, {2.8939, 16.8013}}	{6.33279, 9.0328}	inf	3044.	3160.8	{1505., 5663.}	45.3383	0.183868	newly	1132.	1124.96	{529., 2018.}	45.3815	0.192916	Reff	0.252533	0.219184	{0.205814, 0.239702}	14.6279	ar	91 682.	93 290.9	{93 119., 93 438.}	1.75483
7	R0	{2.63781, 14.3935}	{2.96978, 18.8857}	{{2.96978, 18.8857}, {2.96978, 18.8857}}	{12.5851, 31.2099}	inf	2374.	5782.05	{3092., 9873.}	143.557	0.347494	newly	968.	1867.45	{1013., 3228.}	93.8159	0.249942	Reff	0.252043	0.229353	{0.206251, 0.260004}	12.4333	ar	91 192.	93 821.8	{93 690., 93 953.}	2.8838
8	R0	{2.82391, 16.104}	{2.8939, 16.8013}	{{2.8939, 16.8013}, {2.8939, 16.8013}}	{2.47848, 4.32998}	inf	3239.	4853.73	{1905., 8469.}	71.21	0.206699	newly	1178.	1744.46	{685., 3132.}	70.8195	0.206826	Reff	0.232408	0.237643	{0.20839, 0.267599}	10.9068	ar	92 664.	93 281.1	{93 137., 93 481.}	0.665959
9	R0	{2.80645, 14.6457}	{2.8939, 16.8013}	{{2.8939, 16.8013}, {2.8939, 16.8013}}	{3.11604, 14.7183}	inf	2875.	6410.83	{2920., 9979.}	124.137	0.289012	newly	1160.	2311.56	{1015., 3575.}	103.233	0.249977	Reff	0.229399	0.256469	{0.216608, 0.286091}	14.5754	ar	92 567.	93 243.	{93 075., 93 410.}	0.730297
10	R0	{1.86213, 5.4271}	{2.80387, 10.6536}	{{2.80387, 10.6536}, {2.80387, 10.6536}}	{50.5733, 96.3037}	inf	95.	867.895	{379., 1254.}	813.573	0.87243	newly	92.	443.702	{194., 619.}	382.285	0.594125	Reff	0.460598	0.215444	{0.207655, 0.221225}	53.2251	ar	75 362.	92 555.8	{92 443., 92 696.}	22.8149
11	R0	{2.27349, 14.6338}	{2.96978, 18.8857}	{{2.96978, 18.8857}, {2.96978, 18.8857}}	{30.6265, 29.0553}	inf	2757.	4554.15	{2240., 8607.}	74.5023	0.190703	newly	1137.	1469.97	{730., 2809.}	55.0106	0.171614	Reff	0.351982	0.220553	{0.198233, 0.249818}	39.0199	ar	85 805.	93 848.6	{93 659., 93 990.}	9.37427
12	R0	{2.77261, 14.1894}	{2.85582, 17.2484}	{{2.85582, 17.2484}, {2.85582, 17.2484}}	{3.00114, 21.5583}	inf	2393.	6509.81	{2710., 10 654.}	172.483	0.344275	newly	959.	2341.97	{964., 3788.}	146.31	0.295274	Reff	0.226855	0.282678	{0.220726, 0.300461}	25.6552	ar	92 508.	92 966.4	{92 776., 93 133.}	0.496266
13	R0	{2.65402, 19.3935}	{2.86095, 19.9929}	{{2.86095, 19.9929}, {2.86095, 19.9929}}	{7.79685, 3.09073}	inf	6091.	4865.17	{2933., 7427.}	36.8945	0.189134	newly	2008.	1494.2	{897., 2236.}	38.876	0.209348	Reff	0.292659	0.240436	{0.220865, 0.263179}	18.9071	ar	91 184.	93 007.	{92 865., 93 168.}	1.99929
14	R0	{1.75297, 4.45354}	{2.7943, 13.5962}	{{2.7943, 13.5962}, {2.7943, 13.5962}}	{59.4038, 205.29}	inf	33.	2855.57	{1570., 4907.}	8553.24	1.89378	newly	37.	1213.2	{651., 2091.}	3178.92	1.47206	Reff	0.50468	0.233022	{0.220526, 0.251711}	53.8277	ar	71 263.	92 483.2	{92 365., 92 640.}	29.7773
15	R0	{1.99928, 7.1501}	{2.96307, 14.3219}	{{2.96307, 14.3219}, {2.96307, 14.3219}}	{48.2069, 100.303}	inf	273.	2598.35	{1411., 3758.}	851.778	0.92421	newly	197.	1057.28	{559., 1502.}	436.689	0.675283	Reff	0.411872	0.20332	{0.193429, 0.213341}	50.635	ar	79 602.	93 781.1	{93 609., 93 930.}	17.8125
16	R0	{2.67398, 10.3134}	{2.7943, 13.5962}	{{2.7943, 13.5962}, {2.7943, 13.5962}}	{4.49966, 31.8304}	inf	624.	1816.16	{916., 2745.}	191.176	0.404208	newly	342.	771.657	{409., 1141.}	127.76	0.302251	Reff	0.237957	0.226007	{0.215888, 0.233603}	6.35965	ar	91 289.	92 433.7	{92 258., 92 631.}	1.25388
17	R0	{2.44634, 16.3226}	{2.93978, 19.9817}	{{2.93978, 19.9817}, {2.93978, 19.9817}}	{20.1705, 22.4174}	inf	3997.	7228.74	{3967., 13 484.}	83.1923	0.219433	newly	1461.	2227.88	{1189., 4083.}	60.969	0.17327	Reff	0.317486	0.252837	{0.215339, 0.31538}	24.7904	ar	88 710.	93 601.	{93 456., 93 770.}	5.51344
18	R0	{2.02168, 10.1676}	{2.97157, 19.3547}	{{2.97157, 19.3547}, {2.97157, 19.3547}}	{46.9852, 90.3566}	inf	1489.	6990.84	{3950., 11 589.}	369.499	0.635096	newly	847.	2208.41	{1244., 3689.}	160.733	0.378798	Reff	0.420772	0.240143	{0.209912, 0.286281}	42.9281	ar	80 299.	93 840.8	{93 722., 93 964.}	16.8642
19	R0	{2.89969, 15.009}	{2.8939, 16.8013}	{{2.8939, 16.8013}, {2.8939, 16.8013}}	{0.199677, 11.9415}	inf	2686.	5785.52	{2795., 8464.}	116.87	0.287659	newly	1026.	2073.37	{1021., 3092.}	104.526	0.263082	Reff	0.211938	0.244271	{0.214872, 0.272287}	15.4265	ar	93 349.	93 271.9	{93 073., 93 412.}	0.129657
20	R0	{2.68933, 7.34928}	{2.83858, 10.6973}	{{2.83858, 10.6973}, {2.83858, 10.6973}}	{5.54971, 45.5558}	inf	136.	1176.28	{548., 2119.}	764.911	0.871444	newly	80.	599.607	{282., 1081.}	649.508	0.807554	Reff	0.227975	0.212494	{0.205513, 0.222261}	7.0674	ar	91 574.	92 825.9	{92 646., 92 995.}	1.36711
21	R0	{2.15352, 9.28074}	{2.7943, 13.5962}	{{2.7943, 13.5962}, {2.7943, 13.5962}}	{29.755, 46.4991}	inf	838.	3108.05	{1522., 3971.}	270.889	0.479885	newly	504.	1365.16	{651., 1732.}	171.339	0.333648	Reff	0.358733	0.246738	{0.220945, 0.245004}	37.7931	ar	83 845.	92 455.4	{92 292., 92 591.}	10.2694
22	R0	{2.21953, 9.04387}	{2.99939, 13.7076}	{{2.99939, 13.7076}, {2.99939, 13.7076}}	{35.1363, 51.5679}	inf	694.	3185.48	{1368., 5529.}	359.004	0.555275	newly	414.	1345.56	{560., 2349.}	225.015	0.40266	Reff	0.343938	0.203851	{0.186982, 0.217366}	42.1052	ar	84 856.	94 056.2	{93 897., 94 202.}	10.8421
23	R0	{2.21987, 14.5347}	{2.76795, 19.6041}	{{2.76795, 19.6041}, {2.76795, 19.6041}}	{24.6897, 34.8779}	inf	2885.	5846.65	{2734., 10 329.}	106.746	0.263271	newly	1192.	1837.81	{832., 3363.}	67.178	0.191003	Reff	0.366057	0.271818	{0.237656, 0.323989}	27.5308	ar	85 067.	92 205.5	{92 057., 92 346.}	8.39165
24	R0	{2.84268, 17.9659}	{2.76795, 19.6041}	{{2.76795, 19.6041}, {2.76795, 19.6041}}	{2.62886, 9.11839}	inf	4541.	5706.7	{2879., 8506.}	49.3114	0.165509	newly	1477.	1821.86	{875., 2657.}	49.5914	0.166846	Reff	0.242139	0.280097	{0.237075, 0.293513}	16.5346	ar	92 836.	92 218.6	{92 063., 92 377.}	0.665094
25	R0	{2.52706, 10.3679}	{2.8833, 17.1847}	{{2.8833, 17.1847}, {2.8833, 17.1847}}	{14.097, 65.7491}	inf	746.	5379.28	{2570., 8005.}	621.083	0.800667	newly	432.	1892.88	{907., 2801.}	338.166	0.583203	Reff	0.269107	0.243463	{0.215296, 0.267513}	14.5055	ar	89 623.	93 177.2	{93 027., 93 325.}	3.96578
26	R0	{2.62835, 19.1088}	{2.93978, 19.9817}	{{2.93978, 19.9817}, {2.93978, 19.9817}}	{11.8489, 4.56805}	inf	5501.	6381.93	{2965., 11 079.}	47.5213	0.173659	newly	1758.	1975.01	{924., 3469.}	47.2722	0.177441	Reff	0.292378	0.247239	{0.210165, 0.287422}	23.3372	ar	90 908.	93 614.9	{93 459., 93 730.}	2.97764
27	R0	{2.76831, 19.4155}	{2.99088, 18.9406}	{{2.99088, 18.9406}, {2.99088, 18.9406}}	{8.03992, 2.44598}	inf	4502.	3467.82	{1779., 6580.}	45.2008	0.241006	newly	1418.	1117.82	{596., 2154.}	45.4347	0.237636	Reff	0.252857	0.205172	{0.191386, 0.230358}	18.9933	ar	92 175.	93 988.4	{93 821., 94 161.}	1.96731
28	R0	{2.96609, 16.8903}	{2.7943, 13.5962}	{{2.7943, 13.5962}, {2.7943, 13.5962}}	{5.7918, 19.5029}	inf	3132.	2068.12	{927., 4176.}	54.0571	0.319687	newly	1123.	874.898	{393., 1767.}	51.5478	0.271557	Reff	0.198639	0.227211	{0.214993, 0.244194}	14.3837	ar	94 024.	92 465.7	{92 294., 92 597.}	1.65738
29	R0	{2.17046, 14.39}	{2.76795, 19.6041}	{{2.76795, 19.6041}, {2.76795, 19.6041}}	{27.5283, 36.2342}	inf	3337.	6902.46	{4024., 11 313.}	107.585	0.282344	newly	1397.	2177.82	{1235., 3565.}	61.6827	0.183141	Reff	0.390705	0.281648	{0.2456, 0.334977}	29.5303	ar	84 001.	92 214.4	{92 031., 92 402.}	9.77772
30	R0	{2.88772, 12.4647}	{2.93597, 11.6547}	{{2.93597, 11.6547}, {2.93597, 11.6547}}	{1.67087, 6.49835}	inf	1402.	1307.	{550., 2076.}	46.8837	0.210015	newly	651.	624.972	{267., 956.}	47.2729	0.205476	Reff	0.203498	0.198367	{0.191484, 0.203903}	4.0956	ar	93 273.	93 568.8	{93 431., 93 686.}	0.317393
31	R0	{2.46831, 8.14308}	{2.9194, 10.1535}	{{2.9194, 10.1535}, {2.9194, 10.1535}}	{18.2753, 24.6887}	inf	300.	1072.2	{440., 2091.}	257.401	0.476464	newly	189.	564.797	{244., 1135.}	198.851	0.39837	Reff	0.280894	0.198403	{0.192067, 0.208036}	29.3673	ar	88 762.	93 463.4	{93 327., 93 611.}	5.29668
32	R0	{1.93236, 7.38987}	{2.99939, 13.7076}	{{2.99939, 13.7076}, {2.99939, 13.7076}}	{55.219, 85.4918}	inf	389.	2416.91	{1344., 3563.}	521.314	0.743069	newly	288.	1014.32	{567., 1497.}	252.196	0.494586	Reff	0.437467	0.195632	{0.186472, 0.203179}	55.2808	ar	77 654.	94 052.4	{93 912., 94 159.}	21.1172
33	R0	{2.82251, 17.6954}	{2.86118, 15.5722}	{{2.86118, 15.5722}, {2.86118, 15.5722}}	{1.37006, 11.9986}	inf	4866.	3299.71	{1644., 6041.}	43.1381	0.255489	newly	1670.	1246.2	{643., 2286.}	40.9073	0.228215	Reff	0.244147	0.225669	{0.211012, 0.249495}	9.19057	ar	92 745.	93 025.2	{92 859., 93 189.}	0.302122
34	R0	{1.87083, 12.3994}	{2.76795, 19.6041}	{{2.76795, 19.6041}, {2.76795, 19.6041}}	{47.953, 58.1052}	inf	3095.	5173.17	{2884., 9711.}	71.5404	0.20307	newly	1528.	1629.	{919., 3010.}	38.4674	0.158502	Reff	0.498595	0.262383	{0.237712, 0.308959}	47.3755	ar	76 282.	92 194.4	{91 997., 92 394.}	20.86
35	R0	{2.95779, 16.3003}	{2.86118, 15.5722}	{{2.86118, 15.5722}, {2.86118, 15.5722}}	{3.26629, 4.46679}	inf	3818.	3480.62	{1737., 5747.}	43.0937	0.195961	newly	1428.	1326.88	{683., 2159.}	43.3219	0.192774	Reff	0.209086	0.228378	{0.211527, 0.247292}	9.27462	ar	93 873.	93 011.8	{92 870., 93 164.}	0.917358
36	R0	{2.18718, 14.261}	{2.8833, 17.1847}	{{2.8833, 17.1847}, {2.8833, 17.1847}}	{31.8273, 20.5014}	inf	3033.	1867.17	{850., 3112.}	49.0493	0.304065	newly	1295.	650.667	{308., 1112.}	54.816	0.374431	Reff	0.381269	0.20979	{0.200851, 0.223254}	44.9759	ar	84 379.	93 204.	{92 982., 93 349.}	10.4587
37	R0	{2.44912, 9.31118}	{2.9884, 12.8326}	{{2.9884, 12.8326}, {2.9884, 12.8326}}	{22.0193, 37.8193}	inf	559.	2538.33	{1159., 4854.}	354.085	0.593318	newly	306.	1120.3	{491., 2149.}	266.112	0.499181	Reff	0.289559	0.199281	{0.187821, 0.211907}	31.178	ar	88 442.	93 943.1	{93 762., 94 106.}	6.22004
38	R0	{2.05949, 5.72514}	{2.9884, 12.8326}	{{2.9884, 12.8326}, {2.9884, 12.8326}}	{45.1039, 124.145}	inf	81.	2790.57	{1243., 5207.}	3345.15	1.46605	newly	76.	1235.77	{566., 2258.}	1526.01	1.13953	Reff	0.380141	0.200566	{0.189465, 0.215942}	47.2389	ar	81 587.	93 956.6	{93 806., 94 087.}	15.1613
39	R0	{1.75505, 8.52404}	{2.97157, 19.3547}	{{2.97157, 19.3547}, {2.97157, 19.3547}}	{69.3154, 127.06}	inf	1029.	7141.94	{3849., 10 564.}	594.066	0.795022	newly	719.	2273.54	{1237., 3378.}	216.209	0.451823	Reff	0.527357	0.245055	{0.211189, 0.275613}	53.6562	ar	70 925.	93 840.6	{93 718., 93 987.}	32.3096
40	R0	{2.24724, 13.7861}	{2.93371, 17.1903}	{{2.93371, 17.1903}, {2.93371, 17.1903}}	{30.5472, 24.693}	inf	2420.	3301.03	{1601., 5629.}	52.7397	0.168436	newly	1045.	1153.12	{568., 1951.}	39.9665	0.154207	Reff	0.360008	0.213724	{0.199815, 0.233641}	40.6335	ar	85 257.	93 568.3	{93 385., 93 742.}	9.74856
41	R0	{1.77635, 8.62406}	{2.86118, 15.5722}	{{2.86118, 15.5722}, {2.86118, 15.5722}}	{61.0707, 80.5669}	inf	882.	3262.93	{1783., 5888.}	269.946	0.517671	newly	608.	1240.07	{670., 2217.}	106.349	0.27003	Reff	0.494927	0.224818	{0.21147, 0.246233}	54.5756	ar	72 941.	93 042.6	{92 852., 93 202.}	27.5587
42	R0	{1.80397, 5.4932}	{2.68409, 10.6354}	{{2.68409, 10.6354}, {2.68409, 10.6354}}	{48.788, 93.6103}	inf	102.	743.493	{300., 1166.}	628.914	0.760065	newly	82.	387.149	{160., 584.}	372.133	0.569507	Reff	0.48442	0.236767	{0.230563, 0.242185}	51.1236	ar	73 232.	91 409.4	{91 256., 91 569.}	24.8217
43	R0	{2.83096, 16.7077}	{2.86095, 19.9929}	{{2.86095, 19.9929}, {2.86095, 19.9929}}	{1.05936, 19.6628}	inf	3887.	8818.54	{4736., 16 976.}	127.87	0.313893	newly	1377.	2743.1	{1452., 5337.}	101.373	0.261109	Reff	0.237546	0.287059	{0.23949, 0.408029}	21.3807	ar	92 740.	93 000.2	{92 831., 93 171.}	0.280833
44	R0	{2.73678, 11.6021}	{2.9884, 12.8326}	{{2.9884, 12.8326}, {2.9884, 12.8326}}	{9.19402, 10.6058}	inf	851.	990.318	{534., 1349.}	42.8355	0.147075	newly	393.	434.955	{230., 574.}	41.3178	0.15026	Reff	0.225237	0.186883	{0.180858, 0.19323}	17.1271	ar	92 003.	93 969.3	{93 791., 94 135.}	2.13722
45	R0	{2.14235, 6.98601}	{2.95209, 14.037}	{{2.95209, 14.037}, {2.95209, 14.037}}	{37.7968, 100.93}	inf	172.	2201.43	{1301., 3142.}	1179.9	1.07271	newly	135.	907.776	{532., 1328.}	572.427	0.79376	Reff	0.36707	0.200914	{0.192506, 0.209037}	45.2656	ar	82 974.	93 726.2	{93 583., 93 892.}	12.9585
46	R0	{2.23409, 9.21289}	{2.95376, 15.278}	{{2.95376, 15.278}, {2.95376, 15.278}}	{32.2131, 65.8329}	inf	866.	4758.47	{2196., 7611.}	449.477	0.674303	newly	520.	1881.24	{864., 2972.}	261.777	0.483424	Reff	0.341659	0.242491	{0.200324, 0.244305}	40.8895	ar	85 163.	93 716.3	{93 568., 93 849.}	10.0434
47	R0	{2.12258, 8.15265}	{2.9884, 12.8326}	{{2.9884, 12.8326}, {2.9884, 12.8326}}	{40.7909, 57.404}	inf	433.	3165.36	{1344., 6489.}	631.031	0.767828	newly	280.	1404.38	{609., 2831.}	401.563	0.602448	Reff	0.375293	0.204173	{0.186745, 0.229091}	45.8103	ar	82 690.	93 979.3	{93 845., 94 133.}	13.6526
48	R0	{2.71771, 10.4636}	{2.9884, 12.8326}	{{2.9884, 12.8326}, {2.9884, 12.8326}}	{9.96022, 22.6404}	inf	924.	2995.67	{1274., 5462.}	224.492	0.419521	newly	498.	1329.02	{564., 2409.}	168.528	0.339809	Reff	0.232147	0.203007	{0.18809, 0.222128}	15.4073	ar	91 716.	93 959.2	{93 826., 94 077.}	2.4458
49	R0	{2.44369, 6.66477}	{2.98388, 9.60029}	{{2.98388, 9.60029}, {2.98388, 9.60029}}	{22.1055, 44.0453}	inf	49.	488.406	{245., 766.}	896.747	0.954312	newly	43.	268.359	{131., 425.}	524.092	0.75133	Reff	0.273693	0.183935	{0.179182, 0.187805}	32.7951	ar	88 827.	93 943.4	{93 813., 94 092.}	5.75991
50	R0	{2.77383, 17.9775}	{2.97157, 19.3547}	{{2.97157, 19.3547}, {2.97157, 19.3547}}	{7.12877, 7.66069}	inf	3843.	4936.96	{2836., 7787.}	43.5521	0.149498	newly	1236.	1560.4	{861., 2452.}	42.333	0.147563	Reff	0.244319	0.221036	{0.202483, 0.246729}	11.4956	ar	92 334.	93 836.3	{93 659., 93 982.}	1.62703
